# Proteasomal and lysosomal degradation for specific and durable suppression of immunotherapeutic targets

**DOI:** 10.20892/j.issn.2095-3941.2020.0066

**Published:** 2020-08-15

**Authors:** Yungang Wang, Shouyan Deng, Jie Xu

**Affiliations:** ^1^Institutes of Biomedical Sciences, Zhongshan-Xuhui Hospital, and Shanghai Key Laboratory of Medical Epigenetics, Fudan University, Shanghai 200433, China; ^2^Department of Laboratory Medicine, The First People’s Hospital of Yancheng City, Yancheng 224006, China; ^3^Renji Hospital, School of Medicine, Shanghai Jiao Tong University, Shanghai 200127, China

**Keywords:** Cancer immunotherapy, membrane protein, PROTAC, targeted degradation

## Abstract

Cancer immunotherapy harness the body’s immune system to eliminate cancer, by using a broad panel of soluble and membrane proteins as therapeutic targets. Immunosuppression signaling mediated by ligand-receptor interaction may be blocked by monoclonal antibodies, but because of repopulation of the membrane *via* intracellular organelles, targets must be eliminated in whole cells. Targeted protein degradation, as exemplified in proteolysis targeting chimera (PROTAC) studies, is a promising strategy for selective inhibition of target proteins. The recently reported use of lysosomal targeting molecules to eliminate immune checkpoint proteins has paved the way for targeted degradation of membrane proteins as crucial anti-cancer targets. Further studies on these molecules’ modes of action, target-binding “warheads”, lysosomal sorting signals, and linker design should facilitate their rational design. Modifications and derivatives may improve their cell-penetrating ability and the *in vivo* stability of these pro-drugs. These studies suggest the promise of alternative strategies for cancer immunotherapy, with the aim of achieving more potent and durable suppression of tumor growth. Here, the successes and limitations of antibody inhibitors in cancer immunotherapy, as well as research progress on PROTAC- and lysosomal-dependent degradation of target proteins, are reviewed.

## Introduction

Immune checkpoint blockade therapy has shown promise in restoring the antitumor immune response through activating the immune system^1,2^. Multiple immunocheckpoint pathways have been described, such as those involving cytotoxic T-lymphocyte-associated protein 4 (CTLA-4), V domain-containing Ig suppressor of T-cell activation (VISTA), CD47, OX40, and T cell immunoglobulin and mucin domain 3 (TIM-3)^3–5^. The programed cell death 1 (PD-1)/programed cell death ligand 1 (PD-L1) axis is the most important pathway in terms of clinical therapeutic effects. PD-L1, a type I transmembrane protein containing a short cytoplasmic domain and 2 extracellular domains, is an important immune checkpoint molecule that is commonly expressed on the surfaces of cancer cells^6^ and is encoded by the Pdcd1 gene. The promoter region of Pdcd1 has 2 transcription-factor binding sites (termed conserved regions B and C), which are critical for regulating PD-1 expression. Pdcd1 regulation occurs partly *via* the recruitment of nuclear factor of activated T cells 1 (NFATc1) to a novel regulatory element at the Pdcd1 locus, and it is part of the molecular mechanism underlying the induction of PD-1 in response to T cell stimulation. The interaction between PD-1 on T cells and PD-L1 on cancer cells results in the suppression of tumor-killing activity of T cells, and is a crucial mechanism of tumor immune escape^7^. In addition, tumor-intrinsic oncogenic PD-1 promotes tumor cell proliferation independently of adaptive immunity^8^.

Checkpoint inhibitors promote the anti-tumor immune response by antagonizing suppressive immune checkpoint regulatory pathways. PD-1/PD-L1 blockade therapy restores the effective activation of T-cell-mediated antitumor immunity and has substantial clinical benefits^9^. The production of antibodies targeting PD-1 and PD-L1 has led to the development of drugs targeting these pathways^10^. Multiple anti-PD-1/PD-L1 monoclonal antibodies (mAbs) have been approved as a therapy to treat many types of cancer^11^. However, PD-L1 expression varies significantly among tumor types and stages, and its level changes during therapy^12^. Moreover, the relatively low response rate, acquired resistance, and occasional fatal adverse effects also pose substantial challenges to use of this therapy^13^. The molecular mechanism regulating PD-1/PD-L1 remains largely unknown^14^. Small molecule-mediated inhibition of protein function is a canonical paradigm that enhances the efficacy of most clinical agents^15,16^. However, most small molecule inhibitors of the PD-1/PD-L1 pathway are not yet ready for widespread clinical use, and further preclinical work is required to optimize their formulation and application. Moreover, these small molecule inhibitors can achieve substantial inhibitory effects only when more than 90% of targets are engaged^17^. The high dosing level required can lead to on-target off-tumor effects. Gene knockdown methods based on RNA interference and CRISPR/Cas9 or related strategies have been applied to decrease cellular protein levels and have shown clear therapeutic potential^18,19^. Nevertheless, attaining sufficient concentrations of related agents at the target site is difficult. Safety concerns also arise from off-target effects. Moreover, these agents’ poor metabolic stability causes many adverse effects^20^.

Therefore, PD-1/PD-L1 blockade therapy still faces great challenges that must be overcome. In-depth understanding of the control of PD-L1 expression in tumor cells is required for further improvement of checkpoint blockade therapy. PROTAC, a bifunctional small molecule compound, has been widely studied in the field of anti-cancer drugs and is used as a new therapeutic method^21,22^. The recently reported lysosomal targeting molecules for eliminating immune checkpoint proteins have provided a new direction for targeted degradation of target proteins as crucial anti-cancer targets^23,24^. Here, we primarily review the progress that has been made in degradation strategies for immunotherapeutic targets. These strategies have shown promise in providing an alternative strategy for cancer immunotherapy to achieve more potent and durable suppression of tumor growth.

## Blockade of PD-1/PD-L1 function in cancer immunotherapy

### Monoclonal antibodies

Monoclonal antibodies are a promising strategy for the blockade of PD-1/PD-L1 function. Humanized mAb targeting PD-1/PD-L1 relieves T cell immunosuppression and induces T cell activation, thus restoring the body’s ability to monitor and attack tumor cells. Atezolizumab is the first licensed anti-PD-L1 mAb. Atezolizumab is designed to target PD-L1 through binding to the front beta-sheet of PD-L1^25,26^. Atezolizumab restores the anti-tumor activity of T cells by inhibiting the interaction of PD-L1 with PD-1 on the surfaces of T cells^27^. Pembrolizumab, another humanized anti-PD-L1 mAb, has low affinity for Fc receptors and C1q, and a low likelihood of host immunity stimulation^28^. Pembrolizumab has shown strong anti-tumor activity in phase I clinical trials and is widely used in patients with advanced malignant tumors^29–31^. Nivolumab, a humanized anti-PD-1 IgG4 mAb, binds an N-terminal loop outside the IgV domain of PD-1^32^. Nivolumab has been approved for application in combination with platinum-based chemotherapy^33,34^.

**Figure 1:**
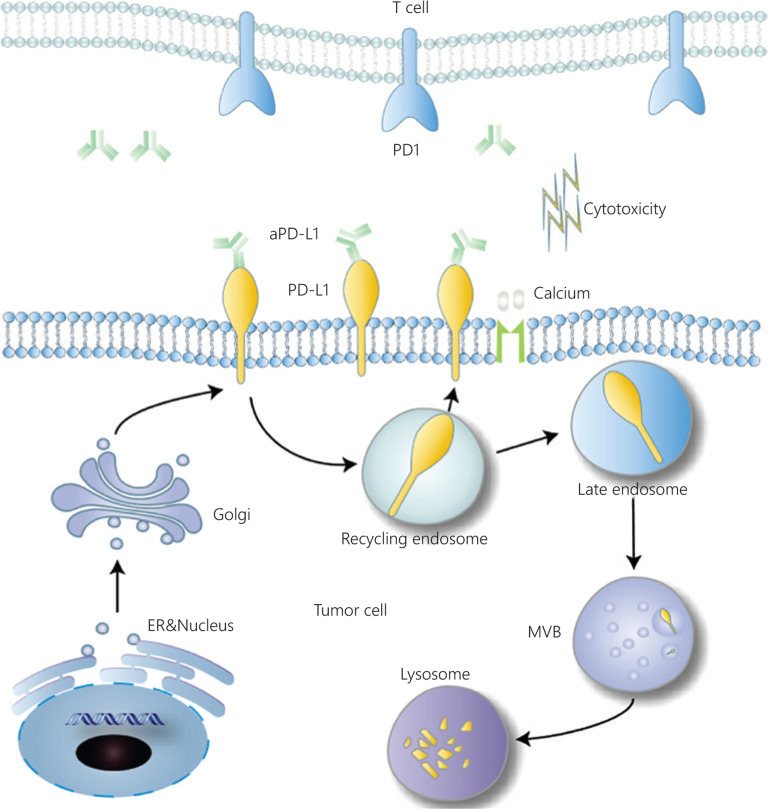
Subcellular transportation of PD-L1 and actions of anti-PD-L1 antibody. The antibody drug binds PD-L1 expressed on the tumor cell surface, thereby blocking its interaction with PD-1. PD-L1 is degraded in the lysosome, in a process relying on several subcellular transport steps from the cell membrane to the endosome, and finally to the lysosome. PD-L1 can also be transported to recycling endosomes, thus decreasing the distribution to late endosomes and lysosomes. PD-L1 is delivered to late endosomes and then sorted to lysosomes *via* multivesicular bodies (MVBs) for degradation.

With the extensive development of clinical treatments, new problems have arisen in the practical use of mAb preparations. Monoclonal antibodies induce the production of anti-mAbs, thus leading to immune-related adverse events (irAEs), such as interstitial pneumonitis, colitis with gastrointestinal perforation, and severe skin reactions^35,36^. In fact, PD-L1 is transported from the plasma membrane into the cytosol and actively redistributed to the plasma membrane, thereby decreasing mAb efficacy, although mAbs can effectively block PD-L1 on the surfaces of tumor cells^8,37^ (**Figure 1**). Emerging evidence indicates that exosomal PD-L1 mediates resistance to immunotherapy by facilitating PD-L1 evasion of the anti-PD1/PD-L1 mAbs^38^. Tumor-derived exosomes carry bioactive PD-L1 molecules on their surfaces that suppress the anti-tumor immune response. This exosomal PD-L1 can enter the blood circulation and inhibit T cells outside the primary tumor tissue, thus causing T cells to lose their anti-tumor ability before reaching the tumor^39,40^. Studies have also revealed the functions of PD-1 and PD-L1 independently of immunosuppression. The intracellular domain of PD-L1 regulates the malignant behaviors of cancer cells and mediates chemoresistance^7^. PD-1 is expressed in a broad range of tumor cells. The cancer-intrinsic PD-1 promotes malignant proliferation by upregulating mammalian target of rapamycin (mTOR) signaling^8^. These functions may explain the unexpected effects of mAbs.

### Small molecule peptide inhibitors

The disadvantages of mAbs limit their application. Although much progress has been made in the development of antibodies against the PD-1/PD-L1 pathway, there is an increasing desire to use small molecules to block the PD-1/PD-L1 axis through dissociating the PD-1/PD-L1 complex. The benefits of using small molecules instead of antibodies include better oral bioavailability, fewer irAEs, better tumor permeability, and lower production costs. The long half-life is a major limitation of mAbs. Small molecule drugs are based primarily on using different therapeutic methods to target the PD-1/PD-L1 pathway. Small molecule inhibitors are more suitable for oral administration and can decrease the target occupancy time by regulating the half-life of the drug, thereby avoiding serious irAEs^41,42^. Tripeptidyl peptidase 1 (TPP1), an active small-molecule peptide, has high affinity for human PD-L1^43^. In a mouse model, TPP-1 has been found to reactivate T cells through blocking the PD-1/PD-L1 interaction and to inhibit tumor growth^44^. Nonylphenol ethoxylate (NP-12), a polypeptide antagonist of the PD-1 signaling pathway, is used as an immunomodulator for cancer treatment^44^. In mouse models of colon cancer and melanoma, NP-12 inhibits PD-1/PD-L1 interaction and suppresses tumor growth and metastasis^45^. CA-170, a small molecule that has been tested in clinical trials, inhibits both the PD-L1 pathway and the VISTA pathway^46^. Sulfamethazine and sulfamethoxazole are small molecules originally produced to inhibit the PD-1/PD-L pathway, and they have been found to rescue PD-1-mediated suppression of IFN-γ secretion^47^. Several other small molecule compounds that inhibit the PD-L1 pathway have been patented^48^. Notably, peptide inhibitors remain in an early stage of development, although they are promising in suppressing immune checkpoints. Furthermore, high drug doses are generally required, thus often leading to undesired adverse effects because of the off-target binding associated with higher drug concentrations^44,45,49^.

## Targeted protein degradation as a promising strategy for drug development

### Small molecule protein proteolysis-targeting chimeras as antitumor agents

Small molecule inhibitors have been used to control cellular protein levels through occupancy-driven pharmacology as the mode of action. However, this strategy only temporarily inhibits the functions of regulatory proteins^50,51^. Finding new models is essential to control cellular protein levels.

**Figure 2:**
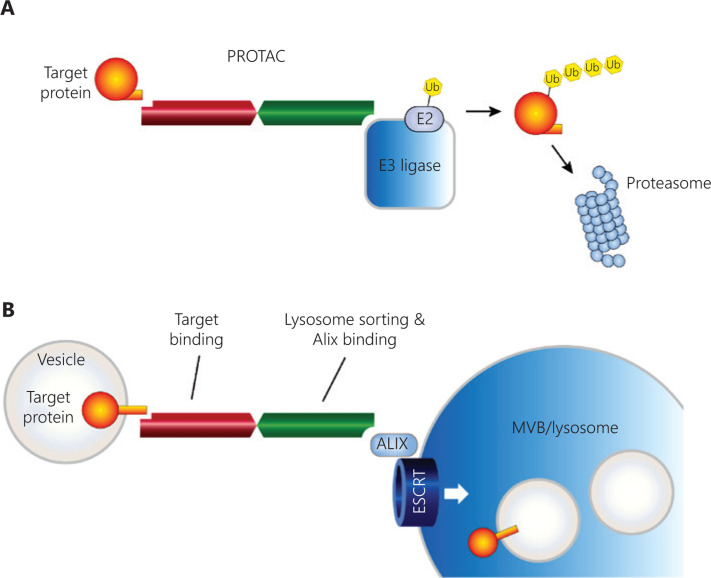
The modes of action of PROTACs and lysosomal targeting molecules. (A) PROTACs can bind target proteins and E3 ligase, thus forming a target protein-PROTAC-E3 ligase ternary complex, which places the target protein and E3 ligase in proximity. Ubiquitin is then transferred from E3 ligase to the target protein. Finally, the target protein is completely degraded through the action of protease. (B) The lysosomal targeting construct contains 2 functional regions: one binds with the target protein, and the other anchors to ALIX and ESCRT for delivery to multivesicular bodies (MVB) and lysosomes.

PROTACs, an attractive new approach for removing proteins by using cellular protein degradation systems to hijack the ubiquitin proteasome system, are playing an increasingly important role in drug discovery^52,53^. PROTACs are activators of ubiquitin ligase whose catalytic properties can be programmed (**Figure 2**)^54^. The heterobifunctional molecules of PROTACs recruit specific target proteins to E3 ubiquitin ligase and then reprogram the enzyme to ubiquitinate the selected target proteins, thus leading to target ubiquitination and degradation. PROTACs also activate ubiquitin ligase through mediating the formation of target protein-PROTAC-E3 ligase catalytic ternary complexes, thus providing a framework for more robust PROTAC designs^55,56^.

In 2001, the first PROTAC was reported to recruit SCFβ-TRCP E3 and subsequently induce degradation of methionine aminopeptidase 2 (MetAp-2)^52^. In 2008, small molecule-based E3 recruitment ligands were invented, and great progress was made in PROTAC technology^57^. Studies indicated the feasibility of developing PROTACs that can enter cells relatively easily^57^. In 2013, mouse experiments provided the first demonstration that phospho-dependent PROTACs (PhosphoPROTACs) inhibit tumor growth *in vivo*^58^. Nonetheless, the peptidic E3 ligase ligands used in PROTACs have hindered their development into more mature chemical probes or therapeutic regimens, because peptidic E3 ligase ligands lead to high molecular weight of the entire PROTAC molecule, thus resulting in poor cell permeability^59^. In 2015, the novel PROTAC HaloPROTAC with incorporated small molecule VHL ligands was reported to successfully degrade HaloTag7 fusion proteins^60^. HaloTag7 is a modified bacterial dehalogenase that covalently reacts with hexyl chloride tags; HaloTag fusion proteins have been widely used to bioorthogonally label proteins *in vivo*. HaloPROTACs resulted in a 90% maximum degradation of GFP-HT7 with a low nanomolar half-maximum degradation concentration^60^. HaloPROTACs inspired the development of future PROTACs with more drug-like properties and have become useful chemical genetic tools^61^ as small molecule proteasome modulators. PROTAC is chimeric with these small molecules, and it forms a bifunctional small molecule compound that can link target proteins and E3 ubiquitin ligase in a ternary complex, thus resulting in target protein degradation through the ubiquitin-protease system^21,22^. Furthermore the development of small molecule-based PROTAC compounds with more drug-like properties has allowed for potent permeable PROTACs to be generated^62,63^. Studies have shown that a PROTAC against P300/CBP-associated factor (PCAF) and general control nonderepressible 5 (GCN5) effectively regulates the expression of multiple inflammatory mediators in macrophages and dendritic cells^64^. To date, a variety of PROTAC variants have been developed, thus laying a foundation for drug advancement. Homo-PROTACs have been developed for auto-targeting of both von Hippel-Lindau (VHL) and cereblon (CRBN)^65,66^.

Additional insights have been gained in the structural basis and target selectivity of PROTACs. PROTACs can be designed to target various proteins of interest, because they are programmable^67,68^. Moreover, the PROTAC technology can escape the resistance mechanisms of inhibitors, including overexpression of target proteins and resistance mutations, by enabling modulation of both the enzymatic and non-enzymatic roles of proteins^69^. PROTAC technology has many advantages, such as low manufacturing cost, low drug dosage, excellent cell permeability, broad tissue distribution, and a strong ability to regulate intracellular targets^70^. PROTACs have already been applied for the degradation of various notable targets, including abelson murine leukemia viral oncogene homolog 1 (ABL)-breakpoint cluster region (BCR) in chronic myeloid leukemia, bromodomain proteins in multiple cancers, and androgen receptor in prostate cancer (details in **Table 1**)^53,71,72^. PD1/PD-L1 have not been selected as target proteins for study, although multiple protein targets have successfully been modulated with PROTAC technology. A study has reported that J22352, a highly selective HDAC6 inhibitor with PROTAC-like properties, decreases the immunosuppressive activity of PD-L1, thus restoring anti-tumor activity in glioblastoma^117^.

### Targeted lysosomal degradation of PD-L1 by PD-LYSO

Although antibodies, small molecule inhibitors, and PROTACs have effects on immunotherapeutic target inhibition, the relatively low response rate and checkpoint blockade resistance have necessitated exploration of the molecular regulatory mechanisms of PD-L1. Studies have revealed the mechanisms that control PD-L1 transcriptional activation and post-translational modifications^118–120^. β transducin repeat containing protein (β-TrCP), cullin 3, and COP9 signaling body 5 (CSN5) control the degradation of PD-L1 through regulating PD-L1 ubiquitination^121,122^. Moreover, targeted blockade of PD-L1 transport from the endoplasmic reticulum to the Golgi apparatus triggers endoplasmic reticulum-related degradation of PD-L1^123^. Studies have found that chemokine-like factor (CKLF)-like MARVEL transmembrane domain-containing proteins 6 and 4 (CMTM6 and CMTM4) increase the stability of PD-L1 through downregulating ubiquitination-dependent degradation and lysosome-dependent proteolysis, thus enhancing the ability of tumor cells to suppress immune responses, and providing a new target for combinatorial immunotherapy^37,124,125^. In fact, the transport between recycling endosomes and lysosomes controls the fate of the PD-L1 protein^37,126^, although the exact mechanism of lysosomal-dependent degradation of PD-L1 is incompletely understood.

Antitumor immunity is enhanced by inhibiting PD-L1, on the basis of the molecular regulation of PD-L1 in tumor cells. Our previous studies have shown that depletion of huntingtin-interacting protein 1-related protein (HIP1R) in tumor cells leads to significant upregulation of PD-L1, thus resulting in the suppression of T cell cytotoxicity^24^. Further research has shown that HIP1R is a regulator of PD-L1 lysosomal degradation that controls PD-L1 homeostasis. HIP1R physically interacts with PD-L1 and transports PD-L1 to lysosomes through a lysosomal targeting signal. HIP1R is an endocytic adaptor protein that contains homology domains responsible for the binding of clathrin, inositol lipids and F-actin^127^. HIP1R binds PD-L1 through its conserved C-terminal domain and uses an intrinsic sorting signal to deliver PD-L1 to lysosomes for degradation^128^. HIP1R targets PD-L1 for lysosomal degradation, thereby enhancing T cell-mediated cytotoxicity, and it is a natural regulator of lysosomal degradation. On the basis of the ‘binding–sorting’ model derived from the molecular roles of HIP1R, we have rationally designed the peptide PD-LYSO, incorporating the lysosome-sorting signal and the PD-L1-binding sequence of HIP1R, and used it to successfully deplete PD-L1 expression in tumor cells^129^. Other researchers have identified SA-49 as a novel regulator of PD-L1 expression from a series of novel aloperine derivatives. They have found that SA-49-induced microphthalmia transcription factor (MITF) translocation functions through activation of PKCα and subsequent suppression of GSK3β activity, thus increasing lysosome biogenesis and promoting translocation of PD-L1 to lysosomes for proteolysis^126^. In breast cancer, another study has identified a disintegrin and metalloproteinase 10 (ADAM10) and ADAM17 as enzymes mediating PD-L1 cleavage. The cleavage generates a free N-terminal fragment and a C-terminal fragment that remains associated with cells but is efficiently eliminated by lysosomal degradation^130^. Researchers are increasingly focusing on exploring more techniques for targeting proteins for lysosomal degradation, including endosome targeting chimeras (ENDTACs), lysosome targeting chimeras (LYTACs)^24^ (**Table 2**).

Thus, the discovery of HIP1R-mediated lysosomal degradation of PD-L1 has provided a potential new route for inhibiting PD-L1. PD-LYSO should be beneficial in the development and optimization of lysosomal targeting strategies as a crucial target for combinatorial immunotherapy.

## Palmitoylation blockade triggers degradation of PD-L1 and PD-1

The intracellular storage and redistribution of PD-L1 to cell membranes minimize the therapeutic benefits^137^. The cytoplasmic domain of PD-L1 is palmitoylated, and this lipid modification stabilizes PD-L1 by preventing its ubiquitination, thereby inhibiting lysosomal degradation^24,138^. Palmitoylation of proteins through linkage to 16-carbon fatty acid palmitate regulates protein localization and function^139,140^. Palmitate is usually associated with cysteine residues through thioester bonds, in a process that may be catalyzed by aspartic acid-histidine-histidine-cysteine (DHHC) palmitosyltransferase^141^. Palmitoylation is a reversible lipid modification of proteins that controls a variety of protein functions, such as transport, activity, stability, and membrane association^142^. Palmitoylation has been shown to regulate the transportation and function of multiple cancer-related proteins^143,144^. Research has indicated that palmitoylation plays an important role in the regulation of PD-L1 protein stability and trafficking and has identified the palmitoyltransferase ZDHHC3 (DHHC3) as the main acetyltransferase required for the palmitoylation of PD-L1^145^. Palmitoylation decreases the lysosomal degradation of PD-L1. The compound 2-bromopalmitate, a small-molecule inhibitor of palmitoylation, blocks the palmitoylation of PD-L1 and effectively induces lysosomal degradation of PD-L1 in tumor cells, thus enhancing the cytotoxicity of tumor-specific T cells^24,138^. The lack of specificity is a major challenge in targeting palmitoylation with existing palmitoylation inhibitors. Apart from the PD-L1-related adverse effects, 2-bromopalmitate might cause adverse effects related to its inhibitory effects on other palmitoylated proteins. PD-PALM, a PD-L1 palmitoylation inhibitor, has been designed to competitively inhibit PD-L1 palmitoylation. The application of PD-PALM decreases PD-L1 expression in tumor cells and enhances T cell activity^24^. Hence, inhibiting palmitoylation of PD-L1 may decrease PD-L1 expression on the cell membrane and deplete its storage capacity in recycling endosomes. Palmitoylation-based targeting methods may provide more powerful and long-lasting inhibitory effects because they inhibit PD-L1 protein levels throughout the cell and therefore may represent a promising therapeutic avenue toward enhancing tumor-specific immunity.

## Challenges and possibilities in future studies

Because most targeted degradation mechanisms rely on intracellular binding and sorting, the cell-penetration ability of therapeutic molecules poses a major challenge. The peptidic nature of PD-LYSO and PD-PALM also makes *in vivo* stability an outstanding challenge, because peptides are generally prone to rapid degradation in the serum, through the action of various enzymes. To achieve a longer half-life, commonly used approaches, such as PEGylation, bovine serum albumin fusion, and Fc fusion, may significantly increase the molecular size and prohibit intracellular delivery. Moreover, potential immunogenic effects should be considered, because peptides may stimulate the generation of neutralizing antibodies *in vivo*. Because of these challenges, developing small molecules to mimic the conformation and functions of peptides is highly preferable and would represent a major step toward successful drug development.

## Figures and Tables

**Table 1:** Molecules that target disease-related proteins for proteasomal degradation

Target	Target classification	Target ligand	Linker	Disease	Molecule/drug name	E3 ligase	Sorting signal	Evidence	References
RIPK2	Kinase	Vandetanib	12 atoms	Multiple cancers	PROTAC_RIPK2	VHL	Small molecule	Preclinical/xenograft	^53^
BCR-ABL	Kinase	Dasatinib	5–10 carbon atoms	Chronic myeloid leukemia	SIAIS178	VHL	Small molecule	Preclinical/xenograft	^73^
		Imatinib, bosutinib, dasatinib	4 different linkers		GMB-101/180/475/651	CRBN, VHL		Cultured cancer cells	^72,74^
EGFR	Kinase	TKIs	Various linkers	Multiple cancers	EGFR-PROTACs	CRBN, VHL	Small molecule	Cultured cancer cells	^75,76^
HER2	Kinase	Lapatinib	Diethylene glycol	Multiple cancers	HER2-PROTAC	VHL	Small molecule	Cultured cancer cells	^71^
c-Met	Kinase	Foretinib	Optimized linker	Multiple cancers	c-Met-PROTAC	VHL	Small molecule	Cultured cancer cells	^71^
TBK1	Kinase	TBK1 ligands	Alkyl ether	Multiple cancers	TBK1-PROTAC	VHL	Small molecule	Cultured cancer cells	^77^
CDK6	Kinase	Palbociclib	Optimized linker	Multiple cancers	Palbociclib-based CDK6 PROTAC	CRBN	Small molecule	Cultured cancer cells	^78^
CDK9	Kinase	Aminopyrazole analogs	Optimized linker	Multiple cancers	CDK9-PROTAC	CRBN	Small molecule	Cultured cancer cells	^79,80^
ALK	Kinase	Ceritinib	Long PEG linker/short carbon linker	NSCLC, DLBCL, ALCL	MS4077/4078	CRBN	Small molecule	Cultured cancer cells	^81^
			Amide linkage		TD-004	VHL		Preclinical/xenograft	^82^
SGK3	Kinase	308-R SGK inhibitor	3 PEG units medium length-ligand	Breast cancer	SGK3-PROTAC1	VHL	Small molecule	Cultured cancer cells	^83^
CK2	Kinase	CX-4945	Alkyl linkers	Multiple cancers	CK2-PROTAC	CRBN	Small molecule	Cultured cancer cells	^84^
ERK1/2	Kinase	Refametinib analogs	Various linkers (optimal 3 atoms)	Multiple cancers	MEK-PROTACs	CRBN, VHL	Small molecule	Cultured cancer cells	^85^
		TCO-tagged inhibitor of ERK1/2 kinases	Optimized linker		ERK-CLIPTAC	CRBN			^86^
FLT3	Kinase	Quizartinib	Optimized linker	Acute myeloid leukemia	FLT3-PROTAC	VHL	Small molecule	Preclinical/xenograft	^87^
PI3K	Kinase	ZSTK474	Linkers longer than hexanamide	Multiple cancers	PI3K-PROTACs	CRBN	Small molecule	Cultured cancer cells	^88^
Akt	Kinase	GDC-0068	10 hydrocarbon	Multiple cancers	INY-03-041	CRBN	Small molecule	Cultured cancer cells	^89^
BTK	Kinase	Ibrutinib	8 atoms	Chronic lymphocytic leukemia	MT-802	CRBN	Small molecule	Cultured cancer cells	^90^
			Optimized linker	Non-Hodgkin lymphomas	L18I	CRBN	Small molecule	Cultured cancer cells	^91^
			Optimized linker	B-cell malignancies	P13I	CRBN	Small molecule	Cultured cancer cells	^92^
Fak	Kinase	Defactinib	Various linkers	Multiple cancers	Fak-PROTACs	CRBN, VHL	Small molecule	Cultured cancer cells	^93^
IRAK4	Kinase	PF-06650833	Two flexible 12 atom linkers	Autoimmune disorders	IRAK4-PROTACs	CRBN, VHL, IAP	Small molecule	Cultured PBMCs	^94^
BRD2/3/4	BET protein	1,4-oxazepines	Linker with an ethynyl group	Multiple cancers	QCA570	CRBN	small molecule	Preclinical/xenograft	^95^
BRD4	BET protein	JQ1	Two ends: a carboxylic acid and an azide group	Multiple cancers	MZ1	VHL	small molecule	Cultured cancer cells	^96^
		OTX015	Flexible polyethylene glycol linker	Burkitt lymphoma	ARV-825	CRBN			^97^
		JQ1	13-atom long PEG-based linker	Multiple cancers	A1874	MDM2			^98^
BRD7/9	BET protein	BrdL1	5 atoms	Multiple cancers	VZ185	VHL	small molecule	Cultured cancer cells	^99^
AR	Nuclear receptor	Aryloxy tetramethylcyclobutanes	Optimized linker	Prostate cancer	ARD69	VHL	Small molecule	Cultured cancer cells	^100^
		Undisclosed	Undisclosed		ARV-110	Undisclosed		Clinical stage I	^101^
		Enzalutamide	Optimized linker		ARCC4	VHL		Cultured cancer cells	^102^
ER	Nuclear receptor	Raloxifene	Linker with a PEG	Breast cancer	ERD-308	VHL	Small molecule	Cultured cancer cells	^103^
		Undisclosed	Undisclosed		ARV-471	Undisclosed		Clinical stage I	^101^
		Bisphenolic-adamantyl system	Alkyl linker		Novel SERDs	Unrestricted	Adamantyl motif	Cultured cancer cells	^104^
TRIM24	Transcription coactivator	IACS-7e	Optimized linker	Acute leukemia	dTRIM24	VHL	Small molecule	Cultured cancer cells	^105^
MetAp-2	Enzyme	Ovalicin	NA	Multiple cancers	MetAP-2-PROTAC-1	SCF(βTRCP)	Polypeptide	Lab research	^106^
Bcl-xL	Transcription factor	A-1155463	Optimized linker	Acute lymphoblastic leukemia	XZ424	CRBN	Small molecule	Cultured cancer cells	^107^
Sirt2	Sirtuin	Triazole-based SirReals	*N*-butyl-2-oxyacetamide linker	Multiple disorders	SirReal-based PROTAC	CRBN	Small molecule	Cultured cancer cells	^108^
HDAC6	Histone deacetylase	Nexturastat A	Optimized linker	Multiple cancers	NP8	CRBN	Small molecule	Cultured cancer cells	^109^
SMAD3	Transcription factor	SMC showing the best binding	Optimized linker	Renal fibrosis	EN300-72284	VHL	Small molecule	Cell models	^109^
PCAF/GCN5	Acetyltransferase	GSK4027	Optimized linker	Multiple inflammatory diseases	GSK983	CRBN	Small molecule	Cultured immune cells	^64^
EED, EZH2, and SUZ12	Polycomb-group proteins	Capped EED ligands	Various linkers	Multiple cancers	EED-targeted PROTACs	VHL	Small molecule	Cultured cancer cells	^110^
Tau	Pathologic protein	YQQYQDATADEQG	GSGS	Alzheimer disease	Keap1-dependent PROTAC	Keap1-Cul3	Polypeptide	Cultured Tau over-expressed cells	^111^
SMARCA2/4	Catalytic subunit	2-(6-aminopyridazin-3-yl)phenols	Polyethylene glycol-based linkers	Multiple cancers	SMARCA2/4-degrading PROTACs	VHL	Small molecule	Cultured cancer cells	^112^
PARP1	Enzyme	Niraparib	Flexible linkers	BRCA1/2 mutant cancers	PARP1-PROTACs	CRBN, VHL, MDM2	Small molecule	Cultured cancer cells	^113^
STAT3	Transcription factor	SI-109	Optimized linker	Multiple cancers	SD-36	CRBN	Small molecule	Cultured cancer cells	^114^
MCL1	Bcl-2 protein	A-1210477	Various linkers	Multiple cancers	MCL1-PROTACs	CRBN	Small molecule	Cultured cancer cells	^115^
Murine double minute 2 protein	Oncoprotein	MI-1061	Optimized linker	Multiple cancers	MD-224	CRBN	Small molecule	Preclinical/xenograft	^116^

**Table 2:** Lysosomal targeting molecules reported in previous studies

Target	Target classification	Binding partner	Disease	Molecule/drug name	Sorting signal	Evidence	Reference
HA-eGFP-HaloTag7 (eGFP-HT7) fusion protein	Fusion protein	CXCR7	Multiple cancers	ENDTAC-1	Small molecule	Cultured cancer cells	^24^
TMED9	Cargo receptor protein	BRD4780	Mucin 1 kidney disease (MKD)	BRD4780	Small molecule	Preclinical/xenografts	^131^
PD-L1	Immune checkpoint	HIP1R	Multiple cancers	PD-LYSO	Peptide	Cultured cancer cells	^129^
		Anti-PD-L1		Anti-PD-L1-M6Pn LYTAC	Glycopeptide	Cultured cancer cells	^132^
Apolipoprotein-E4	Apolipoprotein	LYTAC-Ab1	Neurodegenerative diseases	ApoE4-LYTAC	Glycopeptide	Cultured cells	^132^
EGFR	Growth factor receptor	Cetuximab	Multiple cancers	ctx-M6Pn LYTAC	Glycopeptide	Cultured cancer cells	^132^
CD71	Transferrin receptor	Modified antibody	Multiple cancers	CD71-LYTAC	Glycopeptide	Cultured cancer cells	^132^
RAD6	Ubiquitin E2 conjugating protein	KCMF1	XLID	KCMF1	Peptide	Cultured cells	^133^
EphB3	Receptor tyrosine kinase	Mule	Colorectal cancer	Mule	Small molecule	Cultured cancer cells	^134^
CDK5	Kinase	Tat-CDK5-CTM	Stroke	Tat-CDK5-CTM	Peptide	Preclinical/xenografts	^135^
DAPK1	Kinase	TAT-GluN2BCTM	Stroke	TAT-GluN2BCTM	Peptide	Preclinical/xenografts	^136^
PSD-95	Scaffolding protein	TAT-GluN2B9cCTM	Neural disorders	TAT-GluN2B9cCTM	Peptide	Cultured cells	^136^
α-Synuclein	Pathologic protein	TAT-βsynCTM	Neurodegenerative synucleinopathies	TAT-βsynCTM	Peptide	Cultured cells	^136^
